# Enhanced skin regeneration and therapeutic delivery using novel diamond‐augmented zinc oxide

**DOI:** 10.1111/jocd.16508

**Published:** 2024-07-31

**Authors:** Xinge Diana Zhang, Claudia Teng, Xuefei Bai, Joyce Teng, Suneel Chilukuri, Amy Lewis, Michael H. Gold

**Affiliations:** ^1^ School of Applied Sciences and Engineering Harvard University Cambridge Massachusetts USA; ^2^ B.A.I. Biosciences, Inc. Cambridge Massachusetts USA; ^3^ Department of Dermatology Stanford University School of Medicine Stanford California USA; ^4^ Refresh Dermatology Houston Texas USA; ^5^ Department of Medicine Baylor College of Medicine Houston Texas USA; ^6^ Lewis Dermatology New York New York USA; ^7^ Gold Skin Care Center Nashville Tennessee USA

**Keywords:** collagen synthesis, dermatological therapeutics, diamond‐augmented zinc oxide, skin regeneration, wound healing

## Abstract

**Background:**

Recent advancements in dermatological therapeutics have highlighted the need for treatments that enhance skin regeneration and healing. Diamond‐Augmented Zinc Oxide (ND‐ZnO) technology combines zinc oxide with diamond particles in a unique core‐shell structure, offering a multifaceted approach to overall skin health.

**Aims:**

This study evaluates the efficacy of ND‐ZnO in promoting human dermal fibroblast migration and growth, enhancing total collagen synthesis, and improving transdermal delivery of active ingredients as a daily comprehensive skin regeneration topical therapy.

**Patients/Methods:**

In vitro assays assessed wound healing, collagen production, and skin absorption. Human Dermal Fibroblasts (HDFs) were used in scratch wound assays. Collagen synthesis was quantified using enzyme‐linked immunosorbent assays (ELISA). Permeation tests were performed on reconstructed human epidermal tissues to evaluate niacinamide absorption. Clinical case studies validated ND‐ZnO efficacy in post‐CO_₂_ laser treatments and Actinic Keratosis removal recovery.

**Results:**

ND‐ZnO increased HDF migration by 198% compared to controls. Collagen synthesis assays showed a 71.3% restoration of collagen production in aged HDFs. Skin permeation studies revealed a 203% increase in niacinamide skin absorption with ND‐ZnO. Clinical case studies demonstrated faster and more effective healing post‐ablative CO₂ laser and significant improvements in Actinic Keratosis recovery.

**Conclusions:**

ND‐ZnO technology enhances wound healing, collagen synthesis, and active ingredient delivery, offering substantial benefits for daily skin regeneration and other dermatological applications. This innovative approach holds promise for advancing dermatological therapeutics, providing comprehensive skin care solutions that address both protective and regenerative needs.

## 1 INTRODUCTION

Recent advancements in dermatological therapeutics have increasingly emphasized holistic approaches that not only protect but also rejuvenate the skin. There is a growing demand for innovative treatments that enhance skin regeneration and promote healing, especially following cosmetic procedures or environmental damage.^1^ Traditional skincare solutions often fall short in providing these comprehensive benefits, underscoring the need for novel formulations that address these multifaceted requirements.^2^

Diamond‐augmented zinc oxide (ND‐ZnO) technology represents a significant breakthrough in the field of dermatological therapeutics. This advanced particle combines the well‐known protective properties of zinc oxide with the regenerative and enhancing capabilities of nanodiamonds. The unique structure of ND‐ZnO consists of a porous sphere of zinc oxide crystallites surrounding a central nanodiamond core. This meticulous construction not only enhances the mechanical properties of ZnO but also improves its functional capabilities in dermatological applications. The properties, size, uniformity, and functional characteristics of ND‐ZnO versus conventional ZnO preparations have been previously discussed in detail in our earlier published study.^3^ These particles show a uniform distribution and possess unique porous spheroid structures that cluster uniformly around central nanodiamond particles. These structural properties provide ND‐ZnO with superior performance characteristics compared to conventional ZnO, including enhanced UV protection and significant reduction in reactive oxygen species (ROS) generation.^4^

**FIGURE 1:**
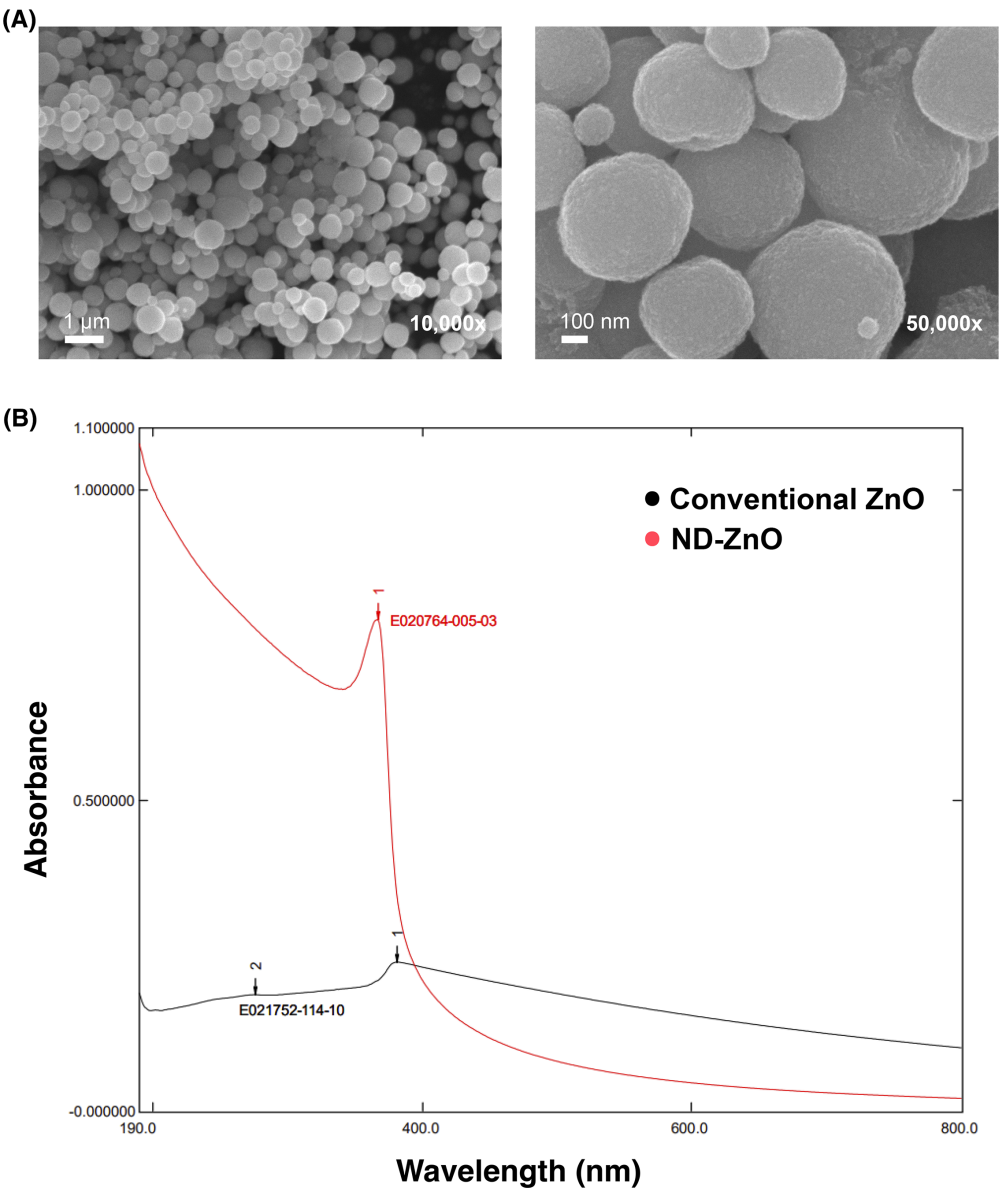
Characterization and Performance of ND‐ZnO Particles (A) Scanning Electron Microscopy (SEM) images of ND‐ZnO particles at various magnifications. The images reveal that these particles are composed of crystallites approximately 10 nm in diameter. Scale bars: 5 μm at 2500x magnification, 500 nm at 50000x magnification, 1 μm at 10000× magnification, and 100 nm at 50000× magnification. (B) UV–Vis absorption spectra comparing ND‐ZnO (red) with conventional ZnO (black). The graph shows that ND‐ZnO has superior UV absorption, particularly in the UVB (290–320 nm) and UVA‐II (315–340 nm) ranges, indicating enhanced protective capabilities against harmful UV radiation. The absorption of ND‐ZnO quickly drops beyond 400 nm, demonstrating its transparency on the skin and lack of white cast typically associated with ZnO sunscreens.

In addition to previous discussions, Figure 1A presents new SEM images of ND‐ZnO. SEM imaging shows the uniform spherical shape of the ND‐ZnO, which may contribute to the enhanced UVB absorption. Furthermore, Figure 1B expands the UV–Vis absorption range comparison of ND‐ZnO and conventional ZnO sunscreens. This demonstrates that ND‐ZnO has enhanced performance in providing protection against harmful UV radiation. The results show unexpectedly high UVB absorption, where ZnO is known to be weak in UVB absorption. This high UVB absorption is significant because even a slight increase in UVB absorption can result in a substantial increase in SPF number.^5^

Another motivation behind developing ND‐ZnO technology stems from the necessity to overcome the limitations of conventional skincare ingredients, which often struggle with poor absorption^6^ and limited efficacy in promoting skin healing and regeneration.^7^ ND‐ZnO technology not only facilitates deeper skin penetration of active ingredients but also enhances their bioavailability, ensuring more effective results.

To evaluate the efficacy of ND‐ZnO in various skin regenerative processes, we conducted experiments focusing on wound healing,^8^ collagen synthesis, and the transdermal delivery of active ingredients.^6^ In our wound healing assays, ND‐ZnO significantly promoted dermal fibroblast growth and migration, resulting in a 198% increase in cell migration compared to the control. Collagen synthesis assays demonstrated that ND‐ZnO effectively prevented and reversed age‐associated collagen degradation, with significant improvements in collagen production. Additionally, permeation tests revealed that ND‐ZnO significantly enhanced the skin absorption of niacinamide, achieving a 203% increase in absorption over a 12‐h period.

Clinical case studies further validated these findings. Patients treated with ND‐ZnO after CO₂‐laser procedures exhibited faster and more effective healing, while a separate case showed significant improvements in a patient with Actinic Keratosis following ND‐ZnO treatment.

These results collectively highlight the transformative potential of ND‐ZnO technology in dermatological therapeutics, offering enhanced wound healing, improved collagen synthesis, and better active ingredient delivery. This paper delves into the detailed findings of our research, providing a robust analysis of the benefits and potential applications of ND‐ZnO in modern dermatology.

## 2 MATERIALS AND METHODS

We conducted in vitro tests included Human Dermal Fibroblast Scratch Wound Assay, Collagen ELISA Assay, and Niacinamide Transdermal Delivery and Skin Permeation Assay. The in vivo tests included clinical case studies involving two patients to evaluate the efficacy of ND‐ZnO cream in wound healing and post‐treatment recovery.

### 2.1 Human dermal fibroblast scratch wound assay

Human dermal fibroblast (HDF) cells were cultured in DMEM, supplemented with 10% fetal bovine serum and 1% penicillin/streptomycin antibiotics. The cells were cultured until around 80% confluency and maintained at 37°C in a 5% CO_2_ humidified atmosphere.

For the scratch wound assay, 2 × 10^5^ cells per well of HDF were plated into a 24‐well plate and incubated for 24 h at 37°C in a 5% CO_2_ humidified atmosphere. The HFD monolayer cell was then scratched by a straight line using a sterile pipette tip to mimic an incision wound. After scratching, PBS was utilized to wash the cells and remove cell debris. The cells were then treated with control and test media. The control medium was DMEM, and the test medium was DMEM with 1.25 μg/mL ND‐ZnO. The cells were incubated for an additional 24 h.

Optical microscopy was employed to analyze five distinct regions along the scratches of each well at baseline and after 24 h. The areas occupied by HDFs were quantified using Image Pro Plus software. The extent of area closure was then calculated and expressed as a percentage in comparison to control cells that remained untreated.

Statistical Analysis: For the scratch wound assay, a *t*‐test was performed to compare the percentage of cell migration between the control group and the ND‐ZnO group.

### 2.2 Collagen ELISA Assay

To assess the impact of ND‐ZnO on collagen synthesis in HDFs, total collagen enzyme‐linked immunosorbent assays (ELISAs) were employed. HDFs were initially seeded in 6‐well plates, each well containing 2.5 mL of DMEM supplemented with 10% FBS. After a 72‐h culture period, or upon reaching 80% confluence, ND‐ZnO at a concentration of 10 μL/mL, PBS, or a positive control comprising 100 μg/mL Vitamin C and 7 μg/mL Vitamin E was introduced for a further 72‐h coculture. Subsequently, collagen levels in the conditioned medium were quantified using an ELISA kit (Abcam), with absorbance measurements at 450 nm via a microplate reader.

To elucidate ND‐ZnO's regenerative influence on HDF collagen synthesis, cells were first exposed to UVA at an intensity of 9 J/cm^2^ to artificially age the fibroblast, followed by the addition of test solutions. This set included a blank control (HDFs with regular cell culture media, no UVA treatment), a negative control (HDFs with regular media, UVA treatment), and an ND‐ZnO group (HDFs with media and ND‐ZnO, UVA treatment).

Conversely, to evaluate ND‐ZnO's protective capacity, test solutions, including a positive control (HDF with media and Vitamins C and E, UVA treatment), were added to each well prior to UVA exposure at 9 J/cm^2^.

Statistical Analysis: The data were analyzed using *t*‐tests to compare specific pairs of groups (e.g., Negative Control vs. Positive Control and Negative Control vs. ND‐ZnO). The *p*‐values for these comparisons were calculated to assess statistical significance.

### 2.3 Niacinamide Transdermal Delivery and Skin Permeation Assay

The diffusion cell was prepared by inserting a magnetic stirrer and placing reconstructed human epidermis tissue in the middle. For the setup, 7.5 mL of 1 × PBS buffer with 4% BSA was added to the bottom section of each cell, and the sampling ports were sealed with parafilm. The experiment involved a set of three parallel cells. The water bath's temperature was maintained at 32°C, and the rotational speed of the magnetic stirrer was set at 180 rpm. Prior to the introduction of the samples, the diffusion cell was pre‐warmed for 10 min. Then, either an ND‐ZnO and 5% niacinamide solution (2 mg/cm^2^) or a 5% niacinamide solution (2 mg/cm^2^) was added to the top section of each cell, with the final mass of approximately 3.53 mg or 3.53 μL, respectively. At intervals of 1, 2, 4, 8, 12, and 24 h, 500 μL of reaction solution was drawn from each cell for analysis, and the cell was immediately refilled with the same volume of PBS buffer with 4% BSA.

The drawn samples underwent a series of processing steps involving dilution with methanol, vortexing for 15–20 s, centrifugation for 10 min at 16000 rpm, and filtration using 0.22 μm PES syringe filters for supernatant collection. The supernatant was then collected for further quantification.

For the calibration sample preparation, a standard solution of 1.0 mg/mL niacinamide in 1 × PBS buffer with 4% BSA was prepared. Each calibration solution underwent a processing protocol similar to that of the permeation test samples, which involved removing BSA to prepare for quantification. The calibration solutions were prepared with the following concentrations: 100, 80, 60, 40, 20, 10, and a blank (0 μg/mL).

Statistical analysis: ANOVA was used to compare mean niacinamide absorption across different time points for the control and ND‐ZnO groups, followed by paired t‐tests each time point on the control and ND‐ZnO groups to determine significant differences at specific time points.

### 2.4 Clinical Case Study

A clinical study was conducted involving two patients to evaluate the efficacy of ND‐ZnO cream in wound healing and post‐treatment recovery. Informed consent was obtained from both patients before and during the study.

#### 2.4.1 Patient 1

This patient, who underwent ablative CO₂‐laser treatment, was provided with ND‐ZnO cream and the standard of care (Aquaphor). The patient applied ND‐ZnO cream to one area of the treated skin and Aquaphor to another area, allowing for a direct comparison of the healing effects between the two treatments.

#### 2.4.2 Patient 2

This patient had Actinic Keratoses removed and was initially dissatisfied with the persistent burn that remained post‐procedure. The patient was supplied with ND‐ZnO cream and applied it to the affected area for 1 week to assess its effectiveness in promoting healing and reducing burn persistence.

## 3 RESULTS AND DISCUSSION

### 3.1 Enhanced human dermal fibroblast migration and growth

**FIGURE 2:**
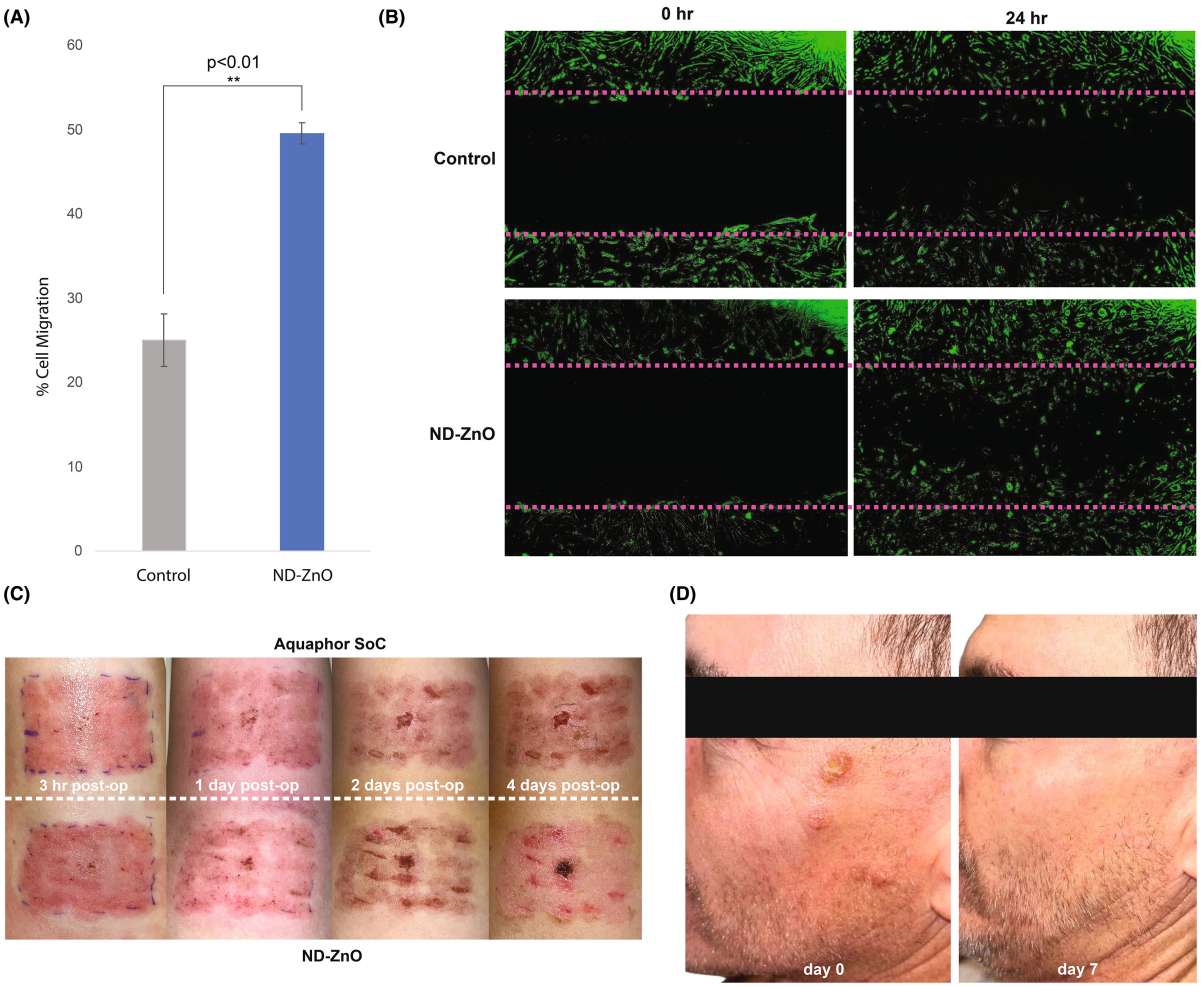
Enhanced Wound Healing with ND‐ZnO. (A) Quantification of cell migration in a wound healing assay. ND‐ZnO significantly increase cell migration compared to the control, demonstrating 198% more dermal fibroblast growth and migration. An independent *t*‐test revealed a significant difference between groups (*𝑝* < 0.01). (B) Fluorescence microscopy images of wound healing assays at 0 and 24 h. The top panels show control samples, while the bottom panels show samples treated with ND‐ZnO. Green fluorescence indicates dermal fibroblasts. The images illustrate the enhanced wound closure and fibroblast migration in the ND‐ZnO treated samples compared to the control. (C) Clinical study images of patient healing post ablative CO₂‐laser treatment. Comparison of wound healing progression over 4 days shows that ND‐ZnO treatment results in faster and more effective healing compared to the standard of care (Aquaphor). (D) Clinical study images showing a patient who had Actinic Keratoses removed and was initially dissatisfied with the remaining, persistent burn. After using ND‐ZnO cream for a week, significant improvement and healing were observed.

The comparative analysis revealed significant migration of HDFs treated with ND‐ZnO, evaluated by quantifying the closure of the scratched area.^8^, ^9^ Across all three plates assayed, the migration of HDFs treated with ND‐ZnO was significantly greater than that of the control group. On average, the wound closure observed in the control group was 25.05% (SD = 3.14%). In contrast, the ND‐ZnO‐treated HDFs demonstrated an average of 49.57% wound closure, which is 1.98 times the HDF proliferation of the control group, with lower variability (SD = 1.23%). An independent *t*‐test revealed a significant difference in wound closure between the control group and the ND‐ZnO, *t* (4) = −12.61, *𝑝* < 0.01. This data underscores the potential of ND‐ZnO as an effective agent for skin regeneration, promoting markedly greater HDF proliferation and migration over a 24‐h period than the control (Figure 2A).

Fluorescence microscopy further corroborated these findings. At both the 0 and 24‐h marks, ND‐ZnO treated samples displayed considerably more fibroblast activity and wound closure than the control samples (Figure 2B). The green fluorescence, indicative of dermal fibroblasts, was markedly more pronounced in the treated samples, demonstrating the potent effect of ND‐ZnO in accelerating cellular processes critical for wound repair.

We conducted two clinical case studies to provide additional evidence of the efficacy of ND‐ZnO in real‐world applications. In the first case, patient who underwent ablative CO₂‐laser treatment, applied ND‐ZnO cream to one area of the treated skin and Aquaphor to another area for 4 days immediately post‐treatment. The results showed faster and more effective healing when treated with ND‐ZnO compared to the standard of care (Aquaphor). Over the 4 days, the ND‐ZnO treated areas exhibited reduced redness and faster tissue regeneration, highlighting the potential of ND‐ZnO in clinical dermatology settings (Figure 2C).

In a separate clinical case study, a patient who had Actinic Keratoses removed experienced significant improvements with ND‐ZnO treatment. Initially dissatisfied with the persistent burn post‐procedure, the patient experienced notable healing after using ND‐ZnO cream for just 1 week. The visual comparison between day 0 and day 7 demonstrated substantial reduction in burn persistence and overall skin recovery (Figure 2D). However, while these results are promising, they are based on individual case studies with a small sample size. Further studies with larger sample sizes are necessary to validate these findings comprehensively.

### 3.2 Improvements in human dermal fibroblast collagen synthesis

**FIGURE 3:**
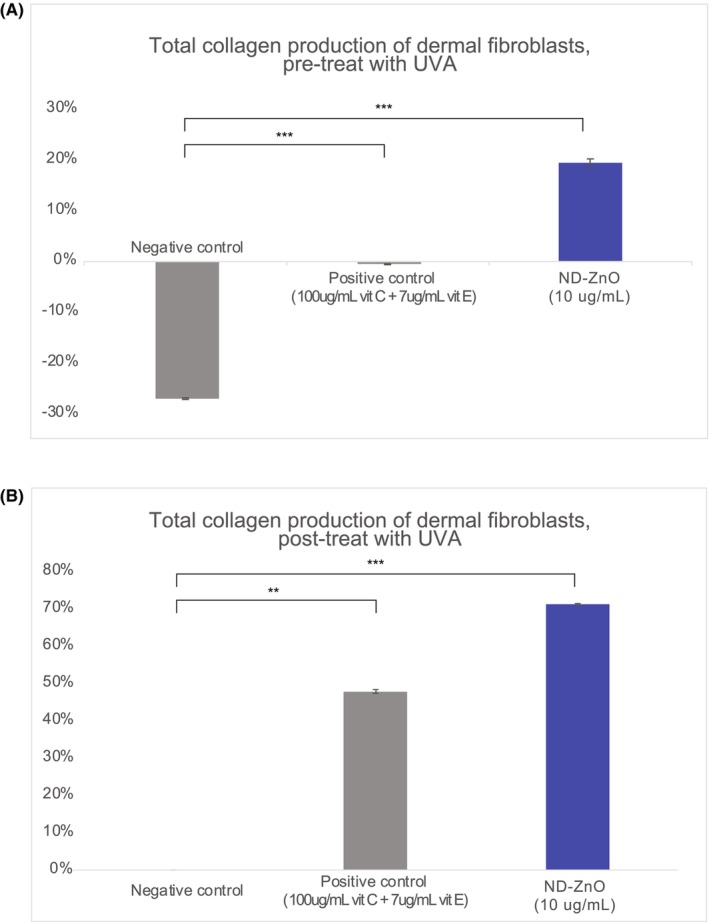
Improvements in Human Dermal Fibroblast (HDF) Collagen Synthesis with ND‐ZnO Treatment. (A) Total collagen production of dermal fibroblasts pre‐treatment with UVA. The graph shows collagen production levels in negative control (no treatment), positive control (Vitamin C + Vitamin E), and ND‐ZnO, *𝑝* < 0.001. (B) Total collagen production of dermal fibroblasts post‐treatment with UVA. The graph shows collagen production levels in negative control (no treatment), positive control (Vitamin C + Vitamin E), and ND‐ZnO (10 μg/mL), *𝑝* < 0.01.

Both the UVA pre‐ and post‐treatment ELISA assay sets demonstrated that ND‐ZnO effectively prevented and reversed age‐associated HDF collagen degradation more efficiently than all other treatment groups. In the UVA post‐treatment evaluation (Figure 3B), ND‐ZnO not only defended HDFs against all UVA‐induced collagen synthesis diminution but also improved HDF collagen synthesis by an average of 19.4% compared to the unaged blank control cells (SD = 0.72%). Significant differences were found between negative control and positive control, *t* (4) = −5.77, =0.0046, and between negative control and ND‐ZnO, *t* (4) = −14.07, *𝑝 =* 0.0002. These findings demonstrate the superior efficacy of ND‐ZnO in promoting collagen synthesis. In comparison to HDFs in the negative control group, which lost 27.1% of their collagen production capability (SD = 0.18%), ND‐ZnO‐treated HDFs demonstrated 46.5% greater collagen production.

In the UVA pre‐treatment analysis (Figure 3A), coculture with ND‐ZnO restored 71.3% of aged HDF's collagen production capacity in comparison to the negative control (SD = 0.31%). Significant differences were observed between negative control and positive control, *t* (4) = −14.45, *𝑝* = 0.0005, and negative control and ND‐ZnO, *t* (4) = −18.35, *𝑝* = 0.0002. These results highlight the enhanced collagen production in cells treated with ND‐ZnO under UVA exposure. ND‐ZnO performed significantly better than the Vitamin C and E positive control group in preventing age‐induced collagen loss, despite the concentration of the Vitamin C and E solution being 10 times that of the ND‐ZnO solution. We notice that Vitamin C and E have some protective effects against degradation but does not have any positive effects on restoring collage production from aged HDFs.

The data indicates that ND‐ZnO is highly effective in stimulating collagen production in aged HDFs, both in preventing collagen degradation and in promoting collagen production restoration in aged cells. This significant improvement in collagen synthesis highlights the potential of ND‐ZnO as a powerful agent in skin regeneration therapies, offering superior protection and restoration of collagen production capabilities in dermal fibroblasts.

### 3.3 Improved transdermal delivery of adjuvant topical niacinamide

**FIGURE 4:**
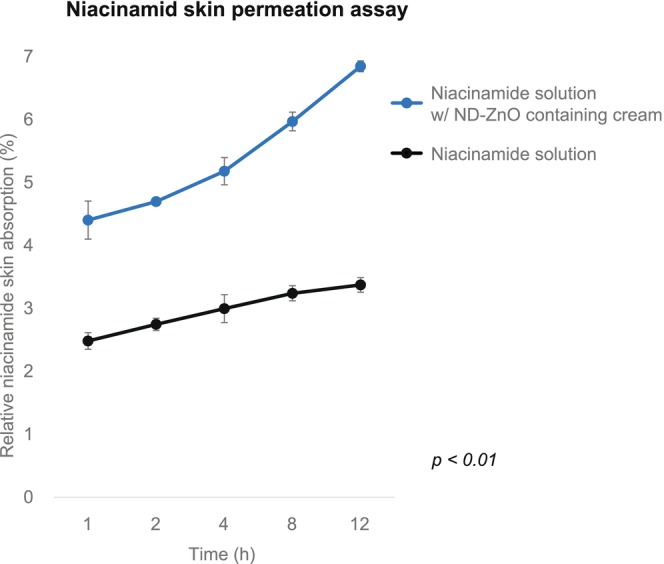
Enhanced Niacinamide Skin Absorption with ND‐ZnO Technology. Relative niacinamide skin absorption (%) over time (hours) comparing a 5% niacinamide solution with a ND‐ZnO + 5% niacinamide solution. The ND‐ZnO containing cream demonstrated over 2× greater skin absorption of niacinamide than the niacinamide solution alone, *𝑝* < 0.01.

The results of the niacinamide skin permeation assay demonstrated that the ND‐ZnO + 5% niacinamide solution significantly enhanced niacinamide absorption compared to the niacinamide solution alone. Over a 12‐h period, the ND‐ZnO containing cream achieved an average relative skin absorption of 6.85% (SD = 0.086*), while the niacinamide solution alone achieved 3.37% (SD = 0.12%), indicating a 203% increase in absorption (Figure 4). ANOVA revealed a significant effect of ND‐ZnO on niacinamide absorption across different time points, *F* (4, 10) = 86.77, *𝑝* < 0.01. Paired *t*‐tests revealed significant differences at 1 h (*𝑝* = 0.0027), 2 h (*𝑝* = 0.0012), 4 h (*𝑝* = 0.0049), 8 h (*𝑝* = 0.0015), and 12 h (*𝑝* = 0.0003).

This substantial improvement in skin absorption underscores the potential of ND‐ZnO technology in maximizing the transdermal delivery of supportive anti‐^6^ therapeutics like niacinamide. By enhancing the skin penetration of niacinamide, ND‐ZnO not only improves the efficacy of niacinamide but also suggests a synergistic effect when combined with other bioactive compounds, amplifying efficacy of topical skincare agents.^6^

## 4 DISCUSSION

There is a growing demand for comprehensive skin regeneration. Recent studies have highlighted the crucial role of advanced skin regenerative technologies in enhancing wound healing,^10^ collagen synthesis,^11^ and overall skin health.^12^ Traditional skincare formulations often fall short in providing these comprehensive benefits, thus underscoring the need for innovative approaches in dermatology.

ND‐ZnO technology represents a transformative advancement in dermatological therapeutics.^3^, ^13^ Our research demonstrates that ND‐ZnO significantly enhances dermal fibroblast growth and migration, improves collagen synthesis, and boosts the transdermal delivery of active ingredients. These findings underscore the potential of ND‐ZnO to revolutionize post‐cosmetic surgery treatments and provide substantial benefits in a wide range of dermatological applications.

ND‐ZnO has demonstrated superior performance in several areas compared to existing treatments. It outperforms Aquaphor in wound healing post‐ablative CO₂ laser treatment, providing faster and more effective recovery. In collagen stimulation, ND‐ZnO surpasses the efficacy of vitamin C and E, enhancing collagen production more effectively. ND‐ZnO also offers better UV filtration than traditional zinc oxide, providing unusual high UVB protection which traditional ZnO falls short, and exceeds known antioxidants in ROS scavenging capabilities.^4^, ^14^ These advantages highlight ND‐ZnO's potential to set new standards in dermatological care.

The implications of ND‐ZnO technology extend beyond enhancing cosmetic outcomes; it offers a versatile platform for improving the efficacy of various therapeutic agents and managing chronic skin conditions. By integrating ND‐ZnO into their practice, dermatologists can offer more effective treatments, promote faster healing, and provide comprehensive skin care solutions that address both protective and regenerative needs.

As we continue to explore the potential applications of ND‐ZnO technology, it is clear that this innovative approach holds great promise for advancing dermatological science and improving patient care. The integration of ND‐ZnO into skincare and therapeutic products has the potential to set new standards in the industry, offering a powerful tool for promoting skin health and achieving better clinical outcomes.

## 5 LIMITATION

Many skin regeneration studies rely on data from small sample sizes due to the challenge of finding comparable patients and the need for extended observation periods. To fully understand the mechanisms of action of ND‐ZnO, additional assays focusing on skin aging‐related biomarkers are needed. Furthermore, to comprehensively elucidate the clinical effects of ND‐ZnO, separate clinical studies with larger sample sizes are needed. These studies should encompass patients with various skin conditions associated with aging and photoaging to validate the broad applicability and efficacy of ND‐ZnO in diverse clinical settings Additionally, it should be noted that both of our clinical case studies were conducted without formal Institutional Review Board (IRB) approval. This limitation should be considered when interpreting the results, and future studies should aim to include formal IRB approval to ensure comprehensive ethical oversight.

## 6 CONCLUSION

ND‐ZnO serves as a multimodal skin regeneration agent by providing high UV absorption, wound healing, collagen restoration, and assisted transdermal delivery of actives such as niacinamide in preclinical and clinical studies. This new science provides a new way in addressing comprehensive skin regeneration needs.

## AUTHOR CONTRIBUTIONS

The conceptualization of this study was carried out by X.B. Data curation was collaboratively managed by X.D.Z., C.T., X.B., J.T., S.C., A.L., and M.H.G., who also contributed to the formal analysis. The methodology was developed by X.D.Z., C.T., X.B., J.T., S.C., A.L., and M.H.G., with resources provided by X.B. Visualization efforts were shared among X.D.Z., C.T., X.B., J.T., S.C., A.L., and M.H.G. The original draft was prepared by X.D.Z. and X.B., while the review and editing process involved contributions from C.T., J.T., S.C., A.L., and M.H.G.

## FUNDING INFORMATION

This work was funded by B.A.I. Biosciences.

## CONFLICT OF INTEREST STATEMENT

C.T. and X.B. are employees of B.A.I. Biosciences, which owns development and commercialization of ND‐ZnO, its formulation and the use thereof.

## ETHICS STATEMENT

The research adhered to the ethical standards stipulated in the most recent iteration of the Declaration of Helsinki. Prior to initiation, all participants were thoroughly informed about the nature and procedures of the study, subsequently providing their written informed consent. These consent documents are securely archived at B.A.I. Biosciences, Inc.

## Data Availability

Research data are not shared.
